# Effectiveness and Methodologies of Virtual Reality Dental Simulators for Veneer Tooth Preparation Training: Randomized Controlled Trial

**DOI:** 10.2196/63961

**Published:** 2025-05-22

**Authors:** Yaning Li, Hongqiang Ye, Wenxiao Wu, Jiayi Li, Xiaohan Zhao, Yunsong Liu, Yongsheng Zhou

**Affiliations:** 1 Department of Prosthodontics, Peking University School and Hospital of Stomatology, National Center of Stomatology, National Clinical Research Center for Oral Diseases, National Engineering Research Center of Oral Biomaterials and Digital Medical Devices, Beijing Key Laboratory of Digital Stomatology, 22 Zhongguancun South Avenue, Haidian District, Beijing, 100081, China, 86 01082195070; 2 The State Key Laboratory of Virtual Reality Technology and Systems, School of Computer Science and Engineering, Beihang University, Beijing, China

**Keywords:** tooth preparation, virtual reality, effectiveness, methodology, dental simulator, veneers, dental education, augmented reality, orthodontics, training, dentistry students, artificial intelligence, simulated reality

## Abstract

**Background:**

Virtual reality (VR) simulators are increasingly used in dental education, offering advantages such as repeatable practice and immediate feedback. However, evidence comparing their efficacy to traditional phantom heads for veneer preparation training remains limited.

**Objective:**

This study aimed to compare the effectiveness of 2 widely used VR simulators (Unidental and Simodont) against traditional phantom heads for veneer tooth preparation training and evaluate the impact of training sequence (simulator-first vs phantom-head-first) on skill acquisition.

**Methods:**

A randomized controlled trial was conducted with 80 fourth-year dental students from Peking University School of Stomatology. Participants were stratified by gender and academic performance, then equally allocated to 8 groups. Groups 1‐3 trained exclusively using Unidental, Simodont, or phantom heads, respectively, while groups 4‐8 followed hybrid sequences combining simulator and phantom-head training. Each participant performed veneer preparations on a maxillary central incisor. Preparations were evaluated by a blinded instructor using a validated 100-point rubric assessing marginal integrity (30%), preparation depth (25%), proximal contour (25%), and surface smoothness (20%). Posttraining questionnaires (100-point scale) compared user perceptions of simulator realism, haptic feedback, and educational value.

**Results:**

There were no statistically significant differences in the preparation quality among groups using different training methods (Unidental: 88.9, SD 3.6; Simodont: 88.6, SD 1.6; phantom heads: 89.4, SD 2.8; *P*=.81) or different training methodologies (simulator-first vs phantom-head-first) (simulator first: *P*=.18; phantom head first: *P*=.09, different sequences of Unidental: *P*=.16; different sequences of Simodont: *P*=.11). However, significant differences were observed between the evaluations of the 2 simulators in terms of realism of the odontoscope’s reflection (Simodont: 55.6, SD 33.7; Unidental: 87.5, SD 13.9; *P*<.001), force feedback (Simodont: 66.2, SD 22.4; Unidental: 50.8, SD 18.9; *P*=.007), and simulation of the tooth preparation process (Simodont: 64.4, SD 16.0; Unidental: 50.6, SD 16.6; *P*=.003). Evaluation results showed no statistical differences between the 2 simulators in display effect (Simodont: 77.43, SD 21.58; Unidental: 71.68, SD 20.70; *P*=.24), synchronism of virtual and actual dental instruments (Simodont: 67.86, SD 19.31; Unidental: 59.29, SD 20.10; *P*=.11), and dental bur operation simulation (Simodont: 63.32, SD 19.99; Unidental: 55.79, SD 19.62; *P*=.16). The Unidental simulator was rated better than the Simodont simulator in terms of the realism of odontoscope’s reflection. In all other aspects, Simodont was superior to Unidental. There was no significant difference in the students’ attitudes towards the 2 simulators (improve skills: *P*=.19; inspire to learn: *P*=.29; will to use: *P*=.40; suitable for training: *P*=.39).

**Conclusions:**

The study found no significant differences in training outcomes between VR simulators and traditional phantom heads for veneer preparation, suggesting that VR technology may serve as a viable alternative or supplementary tool in dental education. However, the absence of significant differences does not imply equivalence, as formal equivalence testing was not performed. Future studies should incorporate equivalence testing and explore cost-effectiveness, long-term skill retention, and adaptability to complex clinical scenarios.

## Introduction

The advent of digitalization has fundamentally revolutionized pedagogical methodologies in dental education. Dental simulators are increasingly being used for skill training in various procedures, including tooth preparation, cavity preparation, periodontal surgery, and implant surgery [1-4]. By leveraging haptic technology, these simulators create realistic clinical environments, offering an innovative paradigm for psychomotor skill development [5]. Unlike traditional phantom heads, dental simulators enable exposure to diversified clinical challenges, allowing students to practice on different tooth positions and complex cases, thereby enhancing their skills [6].

Empirical evidence confirms the complementary value of dental simulators when integrated with traditional phantom heads for skill training [7,8]. However, the synergistic implementation combining both approaches remains underexplored. Currently, well-established dental simulators, including Simodont (Nissin, Netherlands) and Unidental (Unidraw, China), are available for skill training in tooth and cavity preparation. The pedagogical efficacy of these simulators depends on their software and hardware configurations. Consequently, critical knowledge gaps persist regarding comparative effectiveness metrics between virtual and traditional phantom heads, and optimal strategies for integrating virtual reality (VR) dental simulators with traditional head simulators for preclinical dental skills training.

This study aimed to investigate the efficacy of VR dental simulators in the training of tooth veneer preparation, with a focus on exploring their detailed application methodologies. The findings of this study are intended to provide valuable insights and reference for the integration of dental simulators into dental skills training.

## Methods

### Ethical Considerations

This study was reviewed and granted exemption by the Institutional Review Board of Peking University School and Hospital of Stomatology (approval number PKUSSIRB-202498004-免). The research was classified as nonclinical and did not involve interventions or procedures requiring full ethical review. Prior to participation, all volunteers were provided with detailed information regarding the study’s purpose, procedures, and their rights as participants. Written informed consent was obtained from each participant, emphasizing their voluntary participation and the right to withdraw at any time without penalty. All data collected during the study were anonymized to ensure participant confidentiality. Identifiable information was removed, and data were stored securely in password-protected systems accessible only to the research team. Participants did not receive monetary compensation. However, they benefited from experiencing novel training methods for tooth preparation, which could enhance their preclinical skill development. No identifiable images of participants were included in the manuscript or supplementary materials. The Consolidated Standards of Reporting Trails (CONSORT) guidelines were followed (Checklist 1).

### Participants

A total of 80 fourth-year students from Peking University School and Hospital of Stomatology were randomly divided into 8 groups of equal size using a computer-generated randomization sequence stratified by gender and academic performance in prior prosthodontics courses. These students had completed their theoretical courses and were undergoing preclinical prosthodontics training, but had not yet been trained in veneer tooth preparation.

### Evaluation of the Effectiveness of Different Training Methodologies of Dental Simulators

**Figure 1.**
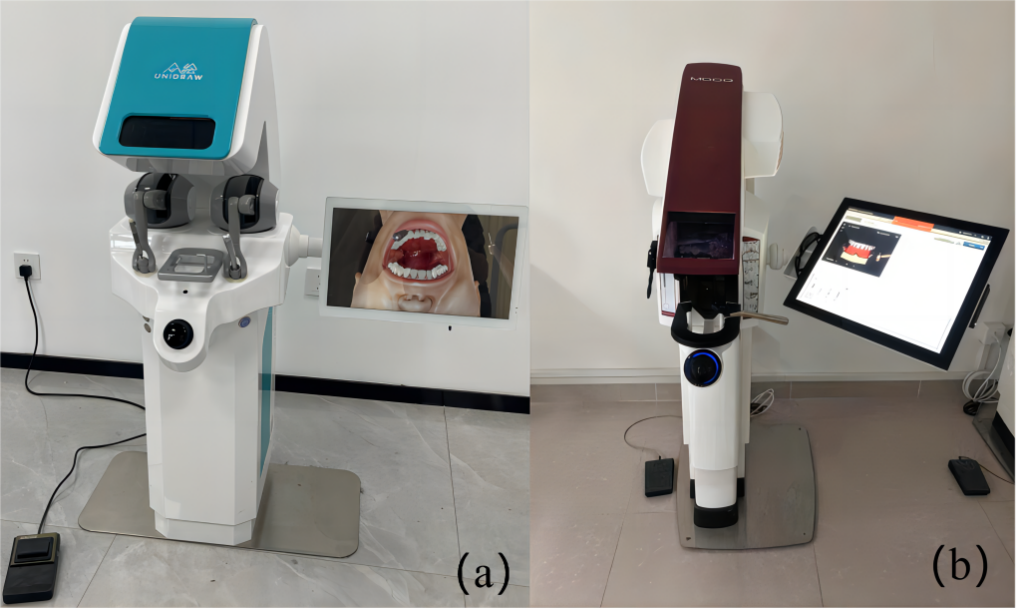
Dental simulators that used in the study. (**A**) Unidental (**B**) Simodont.

**Figure 2.**
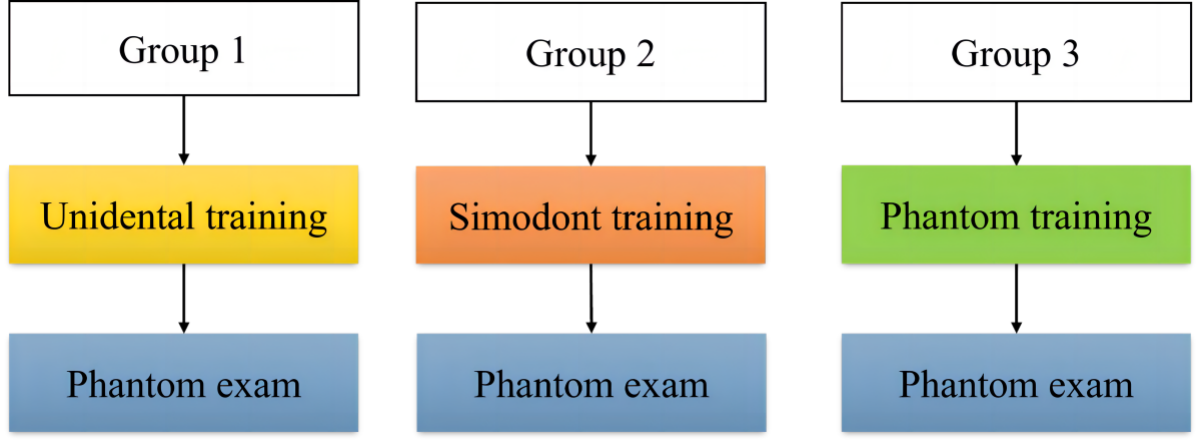
Flow chart for investigating the teaching effectiveness of 2 dental simulators.

Different simulators (Unidental V1.0 and Simodont) (Figure 1) and training methodologies (simulator-first or phantom-head-first) were used to train veneer preparation on the right maxillary incisor. This study had 2 components, as shown in Figures2  3.

**Figure 3.**
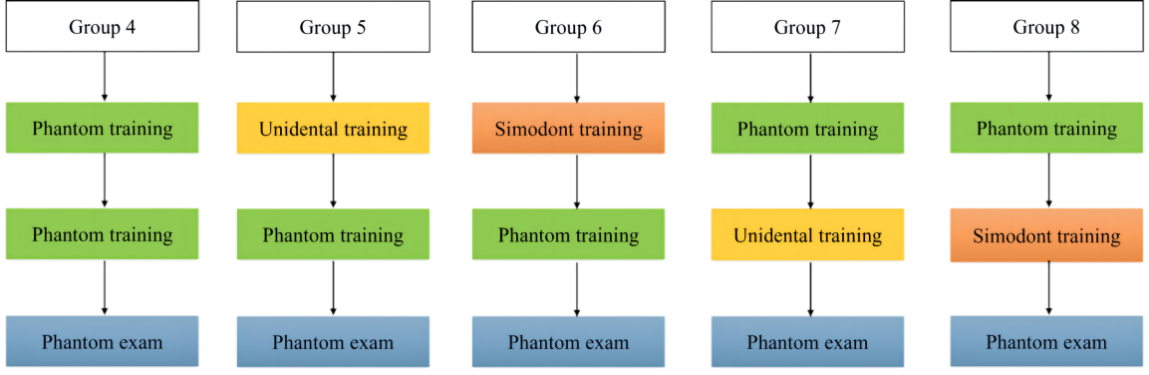
Flow chart for varying training patterns between the 2 simulators.

### First Component

The first component of the study compared the training efficacy of dental simulators and traditional phantom heads. The participants of groups 1, 2, and 3 used Unidental, Simodont, and traditional phantom heads, respectively, for skill training, and were then assessed using traditional phantom heads.

### Second Component

The second component of the study explored the effects of different training methods combining traditional phantom heads and simulators (phantom-first vs simulator-first) in 5 groups. Group 4 served as the control group, with both training sessions conducted on traditional phantom heads. The participants of groups 5 and 6 were initially trained using Unidental and Simodont, respectively, followed by training on traditional phantom heads. Conversely, the participants of groups 7 and 8 began with training on traditional phantom heads, followed by training with Unidental and Simodont, respectively. Each group underwent 2 training sessions before being assessed on traditional phantom heads. All 8 experimental groups performed their training simultaneously.

### Assessment and Evaluation

Participants trained on simulators completed a questionnaire about the 2 simulators after the training. All preparations of the final phantom exam were evaluated by a single experienced instructor (10 y of clinical teaching experience) using a standardized assessment rubric. The rubric comprised four key criteria: (1) marginal integrity, (2) preparation depth, (3) proximal contour preservation, and (4) surface smoothness, with detailed scoring metrics provided in Table 1. The evaluator was blinded to the training method used by each participant. Intrarater reliability was assessed by rescoring 20% of samples, with an intraclass correlation coefficient of 0.89.

The posttraining questionnaire used a 100-point scale to evaluate the simulator feature. Each item (eg, “Realism of odontoscope’s reflection”) required respondents to assign a score between 0 (“Extremely unrealistic”) and 100 (“Indistinguishable from reality”). The total score for each simulator was calculated as the average of all item scores, with higher values indicating better perceived performance.

**Table 1.** Scoring metrics of veneer preparations used in the evaluation process.

Criterion	Scoring range	Description
Marginal integrity	0‐30	Clear, continuous finish line
Preparation depth	0‐25	Uniform 0.5‐0.8 mm reduction
Proximal contour preservation	0‐25	Natural emergence profile maintained
Surface smoothness	0‐20	No grooves/uneven areas

### Statistical Analysis

The sample size was estimated based on the results of a pilot experiment. According to the means and SDs of the 3 samples in the pre-experiment, the required sample size was calculated using the PASS statistical software (NCSS LLC). In the first part of the experiment, based on a test power of 0.8, a sample size of 6 was calculated. For the second part of the experiment, a sample size of 7 was calculated. However, a review of similar studies revealed that a group sample size of 10 was frequently used. Therefore, after thorough consideration, we set the sample size at 10 for each group to satisfy the statistical power requirements.

The Shapiro-Wilk test was used to assess the normality of data. One-way ANOVA was used to analyze differences in veneer preparation examination results on phantom heads among groups 1‐3 and groups 4‐8. Independent samples *t* test was used for questionnaire data that adhered to a normal distribution. For data that deviated from a normal distribution, the Mann-Whitney *U* test was used. SPSS statistics (version 26.0; IBM Corp) was used to analyze the experimental results (*α*=.05).

## Results

### Effectiveness of Different Training Methods for Veneer Tooth Preparation in Groups 1-3

There were no statistically significant differences among groups 1, 2, and 3 in the training results for veneer tooth preparation (*P*=.81, Table 2).

**Table 2.** Virtual veneer preparation results for different dental simulators (*P*=.81).

Group	Training method	Participants, n	Mean (SD)
1	Unidental	10	88.90 (3.57)
2	Simodont	10	88.60 (1.58)
3	Phantom	10	89.40 (2.80)

### Effectiveness of Different Training Methods for Veneer Tooth Preparation in Groups 4-8

There were no statistically significant differences among groups 4‐8 in the training results for veneer tooth preparation (simulator first: *P*=.18; phantom head first: *P*=.09; different sequences of Unidental: *P*=.16; different sequences of Simodont: *P*=.11, Tables3  4).

**Table 3.** Comparison of different sequences of the simulator and phantom head use.

Training pattern and group	Training method	Participants, n	Mean (SD)	*P* value
Simulator first				.18
4	Phantom-Phantom	10	91.10 (1.45)	
5	Unidental-Phantom	10	92.10 (0.88)	
6	Simodont-Phantom	10	91.20 (1.48)	
Phantom head first				.09
4	Phantom-Phantom	10	91.10 (1.45)	
7	Phantom-Unidental	10	91.40 (1.08)	
8	Phantom-Simodont	10	92.20 (0.63)	

**Table 4.** Comparison of different sequences of use for the 2 simulators.

Simulator and group	Training method	Participants, n	Mean (SD)	*P* value
Unidental				.16
4	Phantom-Phantom	10	91.10 (1.45)	
5	Unidental-Phantom	10	92.10 (0.88)	
7	Phantom-Unidental	10	91.40 (1.08)	
Simodont				.11
4	Phantom-Phantom	10	91.10 (1.45)	
6	Simodont-Phantom	10	91.20 (1.48)	
8	Phantom-Simodont	10	92.20 (0.63)	

### Questionnaire Results

**Figure 4.**
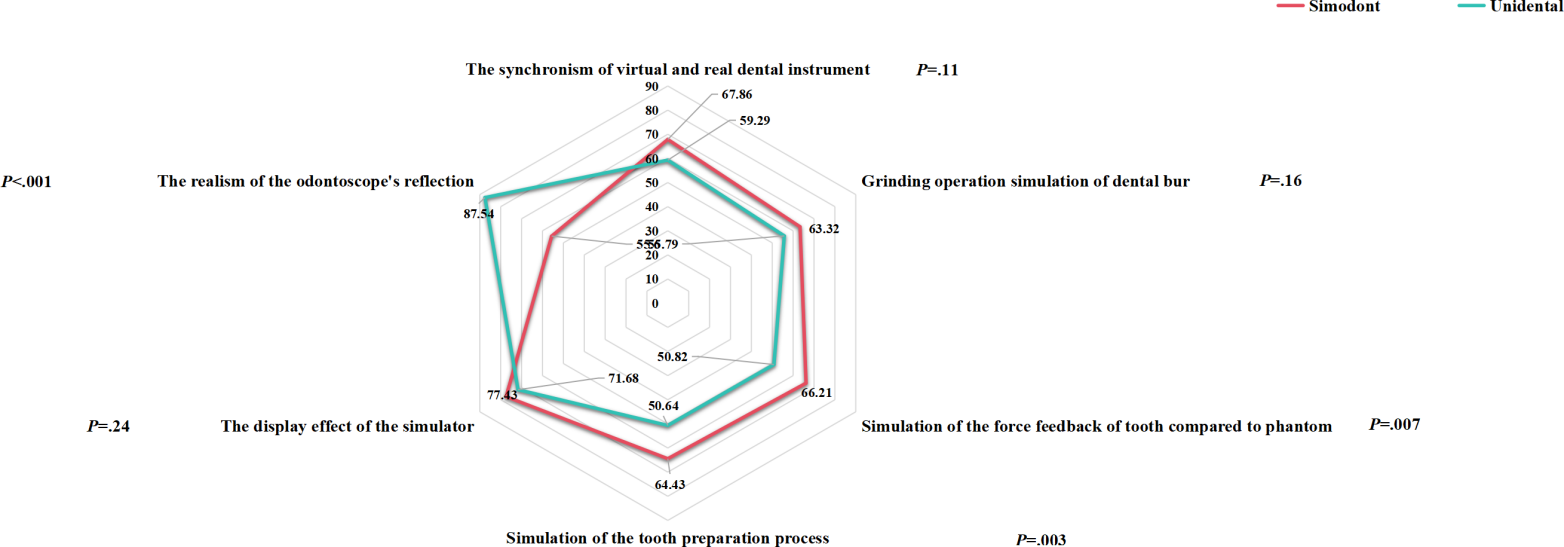
Questionnaire results for the evaluation of the 2 simulators.

In this study, a total of 70 questionnaires were initially collected for analysis. Questionnaires with missing item scores and those with abnormal results (item scores of 0) were excluded, resulting in 56 valid questionnaires, with 28 for each simulator. The evaluation results for the 2 simulators showed no statistically significant differences in display effect (*P*=.24), synchronism of virtual and actual dental instruments (*P*=.11), and the dental bur operation simulation (*P*=.16; Table 5 and Figure 4). However, there were statistical variations between the simulators in terms of the realism of odontoscope’s reflection (*P*<.001), force feedback (*P*=.007), and the tooth preparation process (*P*=.003; Table 5). Unidental outperformed Simodont in the realism of odontoscope’s reflection, while Simodont was superior in all other aspects.

**Figure 5.**
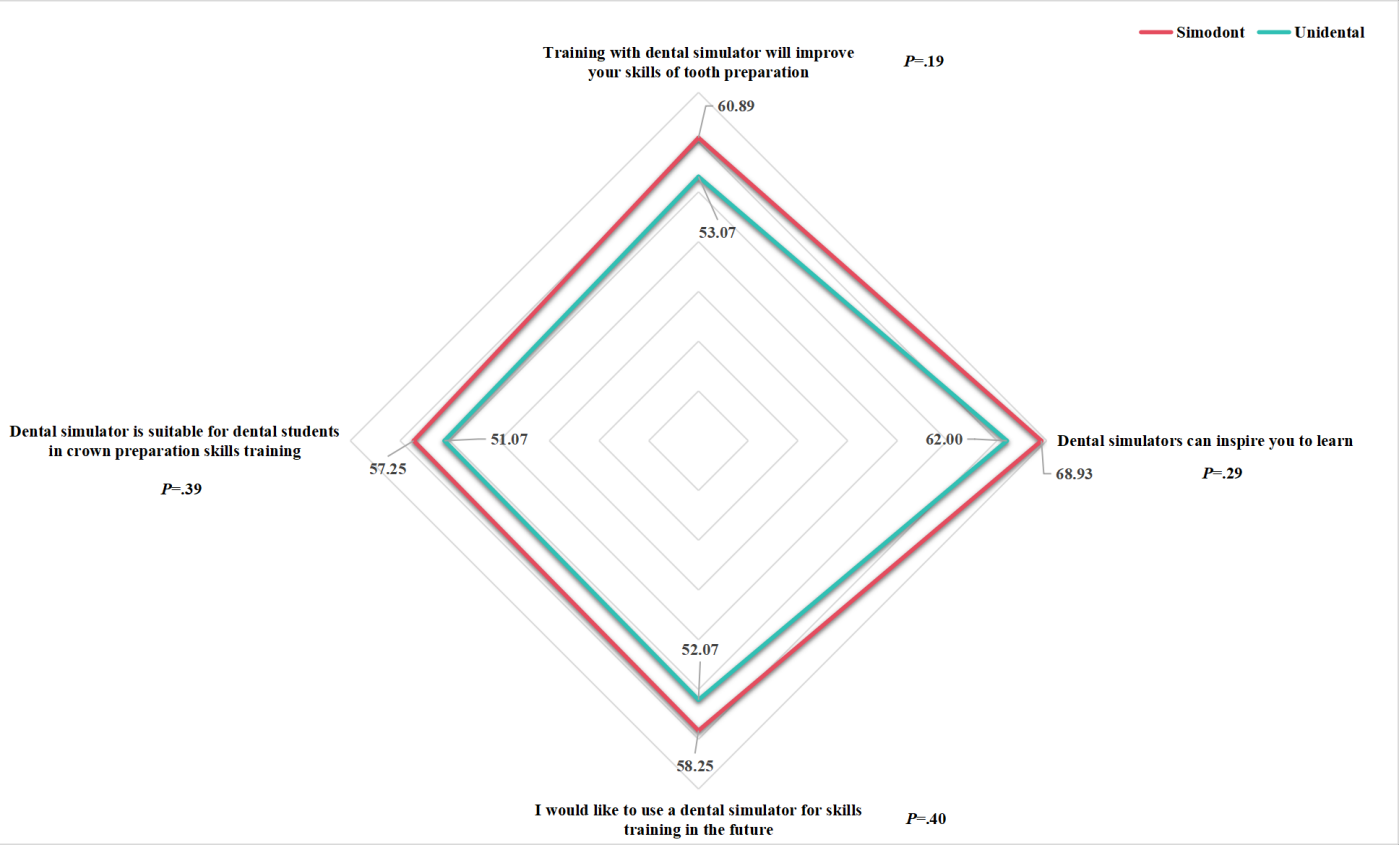
Questionnaire results for volunteers’ attitudes towards the 2 simulators.

Table 6 and Figure 5 present the survey results of students’ attitudes towards the dental simulators, indicating no significant difference between the 2 simulators (improve skills: *P*=.19; inspire to learn: *P*=.29; will to use: *P*=.40; suitable for training: *P*=.39; Table 6).

**Table 5.** Questionnaire results for the evaluation of the 2 simulators. Group 1: Simodont; group 2: Unidental.

Questionnaire item and group	Mean (SD)	*P* value
Simulator display effect		.24
1	77.43 (21.58)	
2	71.68 (20.70)	
Synchronism of virtual and actual dental instruments		.11
1	67.86 (19.31)	
2	59.29 (20.10)	
Dental bur operation simulation		.16
1	63.32 (19.99)	
2	55.79 (19.62)	
Realism of odontoscope’s reflection		<.001
1	55.61 (33.68)	
2	87.54 (13.92)	
Force feedback simulation for tooth compared with phantom		.007
1	66.21 (22.36)	
2	50.82 (18.92)	
Simulation of the tooth preparation process		.003
1	64.43 (15.98)	
2	50.64 (16.62)	

**Table 6.** Questionnaire results for volunteers’ attitudes towards the 2 simulators. Group 1: Simodont; group 2: Unidental.

Questionnaire item and group	Mean (SD)	*P* value
Dental simulator training improved my tooth preparation skills		.19
1	60.89 (23.12)	
2	53.07 (21.02)	
Dental simulators inspired me to learn		.29
1	68.93 (23.62)	
2	62.00 (24.75)	
I would like to use a dental simulator for skills training in the future		.40
1	58.25 (31.38)	
2	52.07 (21.66)	
Dental simulator is suitable for crown preparation skills training of dental students		.39
1	57.25 (31.05)	
2	51.07 (20.88)	

## Discussion

### Principal Findings

The outcomes of the first part of the study demonstrated no significant statistical differences in training efficacy for veneer preparation between the VR dental simulators and traditional phantom heads. To elucidate optimal integration strategies for simulator-phantom head training paradigms, the second part of the study assessed the effectiveness of the simulators and phantom head in various sequences. Data presented in Table 3 reveals that the sequences of using the 2 simulators did not influence the ultimate training outcomes. Comprehensive statistical analyses were conducted to compare analogous sequences of the 2 simulators and different sequences of the same simulator (Table 4). These analyses confirmed that the sequence of the 2 simulators did not impact the final training results, and both simulators achieved comparable training outcomes to traditional phantom head systems in veneer preparation training.

Despite yielding comparable outcomes to traditional methods, simulators provide unique pedagogical advantages. While requiring an initial investment approximately 30‐50 times greater than traditional phantom head units, these advanced systems demonstrate superior long-term cost efficiency through substantial reductions in consumable material expenditures. The elimination of recurring costs associated with resin teeth, dental instruments, and auxiliary materials, coupled with reduced infrastructure requirements for water supply and waste management, contributes to significant operational savings over time [9,10]. VR compensates for the shortcomings of traditional dental skills training by providing repeatable training [11,12], immediate feedback [13-15], and real experiences in simulated environments for students [16,17]. Therefore, dental simulators could be a valuable complement to traditional training.

Since the advent of dental simulators, numerous studies have been conducted to evaluate their efficacy in comparison with conventional phantom-based training methodologies. LeBlanc et al [18] compared the effectiveness of dental simulators by dividing students into 2 groups: the experimental group used simulators for skill training, while the control group used traditional phantoms. The investigation revealed statistically significant superior performance outcomes in the experimental group compared with the control cohort. Buchanan [19] suggested that this might be due to the faster training speed and increased training frequency facilitated by simulator-based education [20]. Urbankova [21] conducted a similar experiment and reached similar conclusions, demonstrating that simulators had a greater impact during the initial phases of skill acquisition. In this study, the training times for the control and experimental groups were identical, resulting in no statistically significant difference in training outcomes for the 2 methods.

Dental simulators have been widely used in preclinical skills training for prosthodontics, particularly in tooth preparation. Both Unidental and Simodont can be used for tooth preparation training. Although the effectiveness of dental simulators in skill training has been previously demonstrated, these studies had some shortcomings. Unidental relies solely on a 2D display, while Simodont also incorporates 3D glasses, providing a 3D experience. Regarding force feedback, Unidental offers soft tissue force feedback, whereas Simodont does not. For veneer tooth preparation, which only requires labial preparation, some volunteers did not use the odontoscope with Simodont because of the lack of soft tissue force feedback. Consequently, Simodont received lower scores than Unidental in the questionnaire survey on the realism of the odontoscope’s reflection. However, Simodont outperformed Unidental in the tooth preparation process, possibly due to its better force feedback experience. Additionally, its 3D display offered better depth perception than the 2D display, allowing students to clearly distinguish the depth of grinding and the smoothness of the preparation surface when using Simodont.

Some studies have indicated that most students feel that the tooth hardness, texture, tactile sensation, and display effects provided by dental simulators are not sufficiently delicate [22]. Although the questionnaire results showed no significant differences in attitudes towards simulator use between the 2 groups, students using Simodont generally exhibited a more favorable attitude compared with those using Unidental. This may be attributed to the superior force feedback and display quality offered by Simodont. These findings highlight the importance of high-quality force feedback and refined display characteristics on user acceptance of the simulator.

Research data on dental simulators in skills training is mostly descriptive and lacks high-level evidence support [23]. Despite technological advancements, existing dental simulators exhibit significant limitations in replicating critical aspects of the oral environment, including but not limited to gingival tissue dynamics, salivary flow, tongue mobility, and essential physiological reflexes such as gag response, coughing, and involuntary head movements [1,17]. In the evaluation of the simulation degree of tooth preparation in this study’s questionnaire, both simulators scored relatively low. This suggests that, besides advancements in force feedback and visual display, improving the realism of the oral environment simulation is also essential. Additionally, both simulators occasionally experienced technical issues during the experiment. Simodont sometimes required rebooting due to initialization failures, while Unidental needed periodic haptic feedback recalibration. So, technical support personnel were present to resolve these issues promptly, ensuring smooth experimental progress. Therefore, the simulators’ inability to replicate critical clinical factors underscores the role of dental simulators as complementary rather than alternative tools for traditional skill training.

Based on these considerations, these studies explored methodologies to combine simulators with traditional phantom heads. Leblanc et al [18] also believed that simulator-based training alone is insufficient for comprehensive skill acquisition. San Diego et al [3] found no difference in the quality of cavity cuts for simple preparations between students trained on dental simulators and those trained on traditional phantom heads. However, students trained on dental simulators performed worse in more complex tasks and in “holding the instruments appropriately.” The first part of this study showed no statistical differences in the training effectiveness for veneer tooth preparation between dental simulators and traditional phantom heads. This may be because veneer tooth preparation is relatively simple and easy to learn.

Therefore, based on the accumulated evidence, dental simulators should not be considered as a replacement for traditional phantom head training in dental education, but rather as a complementary modality within a comprehensive skill acquisition framework. Considering these findings, it is recommended that the dental schools implement an integrated training paradigm that strategically combines the advantages of both simulator-based and traditional phantom head methodologies. This hybrid approach would not only optimize clinical skill acquisition outcomes but also enhance student engagement through diversified training modalities.

### Strengths and Limitations

The main strength of this study was that it accounted for the sequence and types of VR dental simulators when evaluating their training effectiveness. However, there were some limitations. The study did not account for the variability in the rate at which different students acquired proficiency in veneer preparation skills. Individual differences in learning pace were not analyzed, as the primary focus was on evaluating the overall effectiveness of the training methodologies. Consequently, the potential impact of varying learning speeds among participants on the study outcomes remained unexamined. Additionally, due to time constraints, the study did not include separate groups for training using simulators or phantom heads alone. The purpose of this study was to compare the training effect of the VR dental simulator and the traditional phantom head. To avoid the influence of small errors caused by different teachers, a single teacher scored all preparations. However, this approach may have introduced bias due to the teacher’s subjectivity, although an internal consistency analysis was performed. In addition, the sample size of 10 per group, though calculated, may limit statistical power to detect subtle differences. Future studies should recruit larger cohorts to validate these findings.

### Future Directions

Future research directions should focus on 4 key areas to advance the application of VR dental simulator. First, comprehensive pretraining assessments should be implemented to evaluate participants’ visual conditions (eg, normal vision, myopia, astigmatism, color blindness) and their potential impact on simulator performance. Second, technological enhancements should prioritize improving force feedback precision and environmental fidelity, particularly in simulating critical clinical factors such as gingival tissue dynamics, salivary flow, tongue movement, and patient reflexes (eg, gagging, coughing, head movements). Additionally, the integration of cloud-based platforms and remote access capabilities could enable support for remote learning, allowing students to engage in simulator training from diverse locations. This would be particularly valuable in addressing geographical barriers and enhancing accessibility in underserved regions. Finally, expanded validation studies are needed to assess simulator efficacy in complex clinical scenarios, including full-mouth rehabilitation and multidisciplinary treatment planning, as well as its adaptability to hybrid learning models that combine virtual and traditional training methods.

### Conclusions

The study found no significant differences in training outcomes between VR simulators and traditional phantom heads for veneer preparation, suggesting that VR technology may serve as a viable alternative or supplementary tool in dental education. The sequence of simulator use did not significantly influence final competency, providing flexibility in curriculum design. However, the absence of significant differences does not imply equivalence, as formal equivalence testing was not performed. Future studies should address this limitation and explore cost-effectiveness, skill retention, and adaptability to complex scenarios.

## Supplementary material

10.2196/63961 Checklist 1 Consolidated Standards of Reporting Trails (CONSORT)-eHealth checklist.

## Abbreviations

VR: virtual reality
